# Decrease in Infection Rate Following Use of Povidone-Iodine During Transrectal Ultrasound Guided Biopsy of the Prostate: A Double Blind Randomized Clinical Trial

**DOI:** 10.5812/iranjradiol.7561

**Published:** 2012-06-30

**Authors:** Mahyar Ghafoori, Madjid Shakiba, Hamidreza Seifmanesh, Kamal Hoseini

**Affiliations:** 1 Advanced Diagnostic and Interventional Radiology Research Center (ADIR), Tehran University of Medical Sciences , Tehran, Iran; 2 Department of Radiology, Hazrat Rasoul Akram Hospital, Tehran University of Medical Sciences, Tehran, Iran; 3 Department of Urology, Hazrat Rasoul Akram Hospital, Tehran University of Medical Sciences, Tehran, Iran

**Keywords:** Ultrasonography, Biopsy, Prostate, Infection, Povidone-Iodine

## Abstract

**Background:**

Infection after transrectal ultrasound (TRUS) guided biopsy of the prostate is a major and potentially life-threatening problem. Using antibiotic premedication can not completely eliminate infection after biopsy.

**Objectives:**

We performed this study to determine the value of using povidone-iodine in prevention of post biopsy infection.

**Patients and Methods:**

Totally, 280 patients who were referred for TRUS guided biopsy of the prostate were divided randomly into two equal groups. The case group received an intrarectal mixture of povidone-iodine and lidocaine gel before performing biopsy, while the control group received only lidocaine gel. Patients were followed up for 30 days for possible signs of infection including fever, chills and dysuria.

**Results:**

The mean age in the case group was 68.7 ± 7 years and 68.1 ± 7 years in the control group (P = 0.78). Overall, there were signs and symptoms of infection in 78 patients (27.9%), of which 27 (19.3%) were in the case group, while 51 (36.4%) were in the control group (P = 0.001, OR = 2.4, 95% CI = 1.4-4.1).

**Conclusion:**

Simple use of widely available povidone-iodine for cleaning the rectum before TRUS guided prostate biopsy can reduce the infection rate.

## 1. Background

Transrectal ultrasound (TRUS) guided biopsy of the prostate is used widely to detect prostate cancer. This procedure has several minor and major known complications including infection that manifests clinically as fever, chills and dysuria (1). Antibiotic premedication is used routinely to decrease this complication (2, 3, 4), but despite the use of antibiotics, infection develops in many patients with various degrees, from pyuria without clinical symptoms to life-threatening septicemia (5, 6, 7). Different additional methods have been studied for reducing the rate of infection following TRUS guided biopsy including the use of cleansing enema before the procedure (8, 9, 10, 11, 12, 13, 14, 15). However, the results are different. Limited studies are available regarding the use of local antiseptic materials.

## 2. Objectives

As we met decrease in the rate of post biopsy infection with the local use of povidone-iodine antiseptic mixture in our department and considering the fact that these gels and solutions are cheap, safe and easily available, this study was designed to evaluate the efficacy of local use of povidone-iodine as an antiseptic agent in decreasing the rate of infection following TRUS guided biopsies of the prostate.

## 3. Patients and Methods

**Figure 1:**
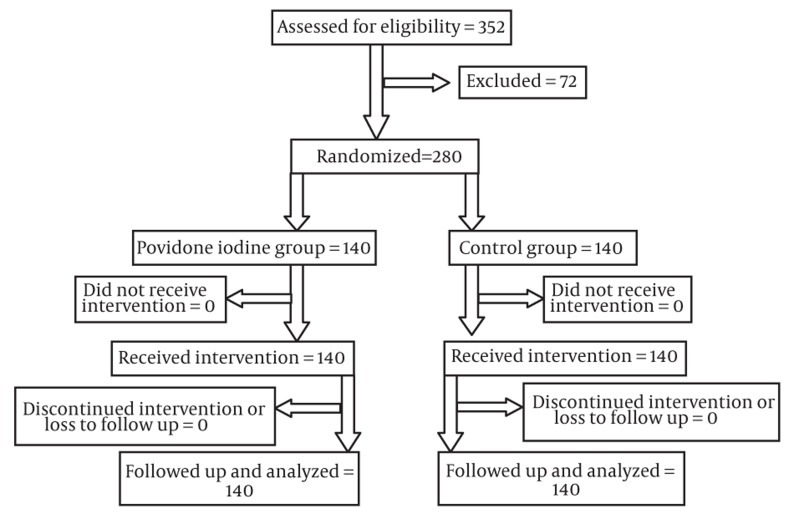
Patients' enrollment, follow up and analysis

The total number of 280 patients who were referred from the Urology Clinic to the Radiology Department of Hazrat Rasoul Akram University-affiliated Hospital from July 2009 to November 2010 was enrolled into the study. All patients were indicated for systematic TRUS guided biopsy of the prostate as investigation for prostate cancer either due to elevated prostate specific antigen (PSA) or abnormal digital rectal exam. Patients who had symptoms of urinary tract infection, patients with diabetes or known immune deficiency, patients who received steroids, patients with indwelling urinary catheters and patients who consumed antibiotics other than our routine protocol were excluded from the study. Patients were divided randomly into two groups, each containing 140 patients (Figure 1). We used four blocking methods for randomization. All the patients as well as the person who followed up the patients were blind to the randomization. Randomization was designed by the researchers; the enrollment process was performed by a nurse. In each patient, 12 core biopsy specimens were obtained by an automatic tru-cut 18 gauge biopsy needle. Antibiotic premedication (ofloxacin 300 mg every 12 hours and metronidazole 250 mg every eight hours) was prescribed to all patients started the day before biopsy and continued for four days. All patients also received rectal bisacodyl suppository 10 mg the night before and on the morning of the biopsy. Before the biopsy in the povidone iodine prescribed group, a mixture containing 50 gr of lidocaine 2% gel with 20 milliliters of povidone-iodine solution was administered into the rectum via a gavage syringe as a lubricant as well as disinfectant. In the control group, only 50 gr of lidocaine gel was administered into the rectum before the biopsy. The mixture and also lidocaine were prepared by a nurse who was not involved in the study. All the patients were blind to the type of gel introduced into the rectum. Biopsy specimens were obtained five minutes after application of gel mixture or lidocaine. Patients were followed-up for 30 days for possible signs of infection including fever (temperature ≥ 38), chills and dysuria. In patients with severe symptoms, blood culture tests were performed to rule out septicemia.

## 4. Results

**Figure 2:**
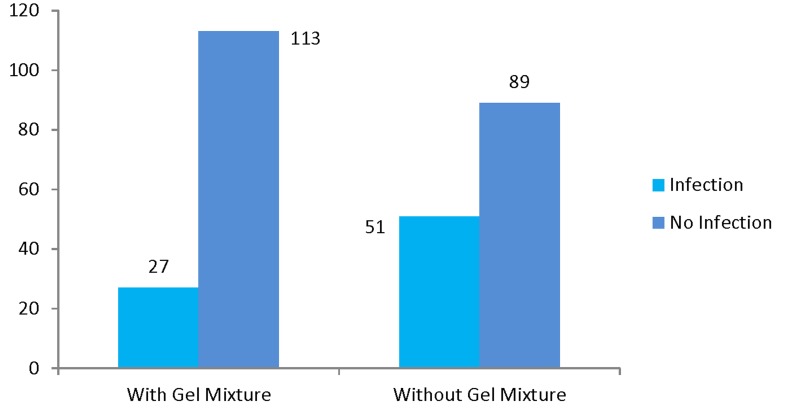
Comparison of infection between the povidone-iodine intervention and control groups

The mean age of patients in the povidone-iodine prescribed group was 68.7 ± 7 years and 68.1 ± 7 years in the control group (P = 0.78). No patients were lost from follow up and we were able to follow all the cases. The mean PSA of the patients was 13.1 ± 12.9. Totally, 78 patients (27.9%) showed signs of infection that needed treatment. Of these, we had 27 cases (19.3%) of infection in the case group, while 51 patients (36.4%) developed infection in the control group (P = 0.001, odds ratio (OR) = 2.4, 95% confidence interval = 1.4-4.1). The comparison of infection between the povidone-iodine prescribed group and the control group has been shown in Figure 2. Among the patients who developed symptomatic infection, 11 patients in the case group (7.9% out of total cases) and 27 patients in the control group (19.3% of total controls) needed hospitalization due to severity of their condition (P = 0.005, OR = 2.8, 95% CI = 1.3-5.9). In two patients of the povidone-iodine prescribed group (1.4% of total cases) and six patients of the control group (4.3% of total controls), septicemia happened that was proved by blood culture test (P = 0.28). No mortality due to biopsy complications happened in neither of the groups. We did not meet any complication or local irritation regarding the local use of povidone-iodine or lidocaine gel.

The statistical analysis of the collected data was performed by SPSS version 16. The values of the variables were given as means ± standard deviations. Normality of variables was checked. Comparison between groups was performed by the independent sample t test and chi square test. A P value lower than 0.05 was considered statistically significant.

## 5. Discussion

Although several complications have been described for TRUS guided biopsy of the prostate (1), the major and most life-threatening of them is infection. It potentially may lead to sepsis (7, 16, 17). Our study shows that enema with a gel mixture containing 50 gram of lidocaine 2% gel with 20 milliliters of povidone-iodide solution as lubricant and disinfectant can significantly reduce the infection rate after biopsy, either prostatitis or more severe life-threatening septicemia, like other studies (9, 10, 11, 14, 18). Although the frequency of septicemia was higher in the control group (three-fold), the difference was not statistically significant. It may be due to the low prevalence of septicemia in the two groups regarding the total sample size. In a similar study, Park et al.(14) studied the effect of povidone-iodine suppository on infectious complication after TRUS guided biopsy of the prostate. Totally, infectious complications occurred in 6.6% of the rectally non prepared group while in the rectally prepared group, only one patient was involved (0.3%). Although they found a lower rate of infection in each of the study groups compared with our study, their results are in agreement with our study regarding this fact that the control group faced more infections than the povidone-iodine prescribed group. Acute prostatitis is a major infectious complication of TRUS guided biopsy of the prostate and E.coli has been reported to be the main cause (19). Park et al. (14) suggested that melting povidone-iodine into the rectum may decrease the bacterial colony count (including E.coli). Another study by Kanjanawongdeengam et al. (11) on 100 patients has reported similar results to our study. They found that sterilization of the rectum before TRUS guided prostate biopsy can reduce clinical infections. Huang et al. (9) also reported that bowel preparation before prostate biopsy using povidone-iodine (in addition to antibiotic prophylaxis) is an effective way to reduce the infection. There are two older studies that are in agreement with our study (10, 13). A reverse result was reported by Koc et al. (12). They enrolled 180 patients and used a needle that was washed with povidone-iodine for the povidone-iodine prescribed group. Considering infection, there was no statistically significant difference between the two groups. They concluded that washing the biopsy needle with povidone-iodine cannot reduce the infection rate. It may be due to insufficient sterilization of the washed needle. They only used a washed needle with povidone-iodine while we introduced povidone-iodine into the rectum. A very recent study (15) has reported that prebiopsy enema does not have a significant effect on reducing the overall complication rate. However, they found that the incidence of infection or sepsis in the prebiopsy enema group was lower than the rectally non-prepared group (P > 0.05). Using antibiotic premedication is still the best way for prevention of infectious complications of TRUS guided biopsy of the prostate. However, use of povidone-iodine solution is found to be a simple and cheap way to further reduce infectious complications.

A considerable percent of patients develop infection following TRUS guided prostate biopsy despite oral antibiotic premedication. The difference in the rate of infection between the two groups was significant and adding povidone-iodine to lidocaine gel may decrease the rate of infection.

## References

[1] Raaijmakers R, Kirkels WJ, Roobol MJ, Wildhagen MF, Schrder FH (2002). Complication rates and risk factors of 5802 transrectal ultrasound-guided sextant biopsies of the prostate within a population-based screening program. Urology.

[2] Ho HS, Ng LG, Tan YH, Yeo M, Cheng CW (2009). Intramuscular gentamicin improves the efficacy of ciprofloxacin as an antibiotic prophylaxis for transrectal prostate biopsy. Ann Acad Med Singapore.

[3] Munoz Velez D, Vicens Vicens A, Ozonas Moragues M (2009). [Antibiotic prophylaxis in transrectal prostate biopsy].. Actas Urol Esp.

[4] Yang M, Zhao X, Wu Z, Xiao N, Lu C (2009). Meta-analysis of antibiotic prophylaxis use in transrectal prostatic biopsy. J Cent South Uni Med Sci.

[5] Al-Otaibi MF, Al-Taweel W, Bin-Saleh S, Herba M, Aprikian AG (2004). Disseminated intravascular coagulation following transrectal ultrasound guided prostate biopsy. J Urol.

[6] Hasegawa T, Shimomura T, Yamada H, Ito H, Kato N, Hasegawa N (2002). [Fatal septic shock caused by transrectal needle biopsy of the prostate; a case report].. Kansenshogaku Zasshi.

[7] Kato K, Suzuki K, Sai S, Senda M, Murase T (2001). [A case of septic shock and disseminated intravascular coagulation following transrectal prostatic biopsy].. Nihon Hinyokika Gakkai Zasshi.

[8] Carey JM, Korman HJ (2001). Transrectal ultrasound guided biopsy of the prostate. Do enemas decrease clinically significant complications?. J Urol.

[9] Huang YC, Ho DR, Wu CF, Shee JJ, Lin WY, Chen CS (2006). Modified bowel preparation to reduce infection after prostate biopsy. Chang Gung Med J.

[10] Jeon SS, Woo SH, Hyun JH, Choi HY, Chai SE (2003). Bisacodyl rectal preparation can decrease infectious complications of transrectal ultrasound-guided prostate biopsy. Urology.

[11] Kanjanawongdeengam P, Viseshsindh W, Santanirand P, Prathombutr P, Nilkulwattana S (2009). Reduction in bacteremia rates after rectum sterilization before transrectal, ultrasound-guided prostate biopsy: a randomized controlled trial. J Med Assoc Thai.

[12] Koc G, Un S, Filiz D, Akbay K, Yilmaz Y (2010). Does Washing the Biopsy Needle with Povidone-Iodine Have an Effect on Infection Rates after Transrectal Prostate Needle Biopsy?. Urol Int.

[13] Melekos MD (1990). Efficacy of prophylactic antimicrobial regimens in preventing infectious complications after transrectal biopsy of the prostate. Int Urol Nephrol.

[14] Park DS, Oh JJ, Lee JH, Jang WK, Hong YK, Hong SK (2009). Simple use of the suppository type povidone-iodine can prevent infectious complications in transrectal ultrasound-guided prostate biopsy. Adv Urol.

[15] Zaytoun OM, Anil T, Moussa AS, Jianbo L, Fareed K, Jones JS (2011). Morbidity of prostate biopsy after simplified versus complex preparation protocols: assessment of risk factors. Urology.

[16] Hoshi A, Nitta M, Hongoh S, Hanai K, Nishikawa Z, Kobayashi Y (2006). [Sepsis following transrectal prostate biopsy: a report of 2 cases and reviewed similar cases in Japan]. Hinyokika Kiyo.

[17] Simsir A, Kismali E, Mammadov R, Gunaydin G, Cal C (2010). Is It Possible to Predict Sepsis, the Most Serious Complication in Prostate Biopsy?. Urol Int.

[18] Chiang IN, Chang SJ, Pu YS, Huang KH, Yu HJ, Huang CY (2007). Major complications and associated risk factors of transrectal ultrasound guided prostate needle biopsy: a retrospective study of 1875 cases in taiwan. J Formos Med Assoc.

[19] Kim SJ, Kim SI, Ahn HS, Choi JB, Kim YS (2010). Risk factors for acute prostatitis after transrectal biopsy of the prostate. Korean J Urol.

